# Acute coronary syndrome in an 8-year-old child with familial hypercholesterolemia: a case report

**DOI:** 10.1186/s13256-022-03488-3

**Published:** 2022-07-26

**Authors:** Nan Wang, Ying Wei, Gang Zhou, Yue Zhang, Jiyang Song

**Affiliations:** 1 Department of Cardiology, Gansu Provincial Hospital, Lanzhou, 730000 Gansu Province China; 2 Department of Paediatrics, Soochow University, Suzhou, 215123 Jiangsu Province China

**Keywords:** Familial hypercholesterolemia, LDLR, Acute coronary syndrome, Filtration plasma

## Abstract

**Background:**

Familial hypercholesterolemia (FH) is the most commonly inherited metabolic disease and has an autosomal dominant mode of inheritance. Patients with FH usually present with high levels of low-density lipoprotein-cholesterol, xanthomas and early coronary artery disease. Children with FH are subject to developing early and enhanced atherosclerosis, leading to an increased risk of coronary events. We report here an 8-year-old patient with acute coronary syndrome (ACS) who was diagnosed with homozygous FH (HoFH) due to mutations in the low-density lipoprotein receptor (LDLR) gene and subsequently treated with percutaneous transluminal coronary angioplasty (PTCA) after filtration plasma. To the best of our knowledge, this patient is the youngest case of HoFH treated with filtration plasma followed by PTCA reported to date.

**Case presentation:**

An 8-year-old Asian patient was admitted to Gansu Provincial Hospital presenting symptoms of ACS. Laboratory tests showed that the patient’s cholesterol and low-density lipid levels were extremely high. An electrocardiogram (ECG) revealed sinus arrhythmia and electric axis deviation to the right, but the ECG was roughly normal. Multiple cardiac function abnormalities were diagnosed on the ECG. Multiple sites of coronary artery stenosis were determined by computed tomography angiography. DNA sequencing of exons showed a C-to-A substitution at nucleotide 126 in exon 2, resulting in a LDLR mutation in the patient and seven other family members. Following combination treatment with lipid-lowering drugs, anti-thrombosis drugs, filtration plasma and PTCA, the patient’s symptoms were significantly improved and the patient discharged.

**Conclusion:**

We report the rare case of a patient with ACS attributable to HoFH who was treated with PTCA following filtration plasma. The patient was shown to have clinically diagnosed and molecularly confirmed HoFH that resulted from a mutation in the LDLR gene. Children with HoFH have higher risk of early coronary events and death from myocardial infarction due to premature atherosclerosis than adults. Earlier intervention and treatment will bring great benefits to the long-term survival of pediatric patients.

## Background

Familial hypercholesterolemia (FH) is a genetic disorder caused by mutations in the genes related to the low-density lipoprotein receptor (LDLR) pathway, and is transmitted by autosomal dominant inheritance [1, 2]. The main pathogenic genes encoding LDLR are apolipoprotein B (ApoB), proprotein convertase subtilisin kexin-9 (PCSK9) and low-density lipoprotein receptor adaptor protein 1 (LDLRAP1) [3]. Patients with FH have extremely high levels of low-density lipoprotein-cholesterolemia (LDL-C), leading to progressive atherosclerosis from a young age and resulting in a high risk of coronary artery disease (CAD) [1, 4]. The prevalence of heterozygous FH (HeFH) is reported to be 1:100–250, but only a minority of such patients are diagnosed/treated [5, 6]. In contrast, the prevalence of homozygous FH (HoFH) is much lower. HoFH is defined as the presence of two pathogenic mutations in the two alleles of the causative genes. Here, we report an 8-year-old Asian patient with HoFH who was admitted to our hospital with symptoms of acute coronary syndrome (ACS). Physical examinations, laboratory tests and genetic screening revealed that the patient had typical HoFH symptoms. Cardiac coronary artery stenosis was further determined by cardiac angiography (CAG). The patient was immediately treated with a combination of lipid-lowering, anti-thrombosis, double filtration plasmapheresis and percutaneous transluminal coronary angioplasty (PTCA) therapies. The patient was discharged when the myocardial ischemia symptoms were successfully controlled.

## Case presentation

**Fig. 1:**
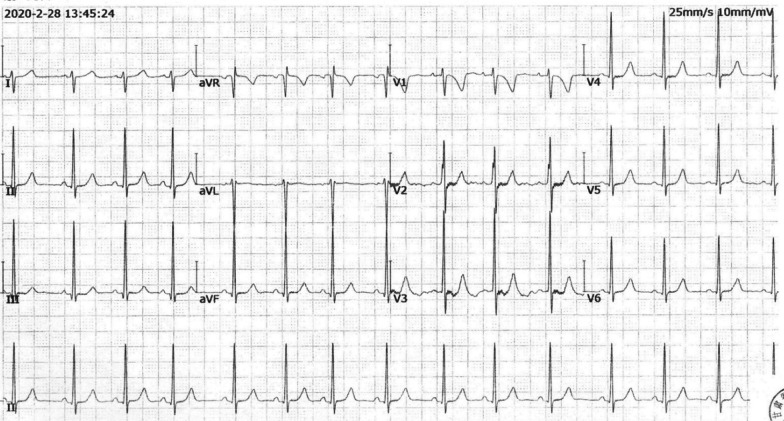
12-lead electrocardiograms at the general hospital.

**Fig. 2:**
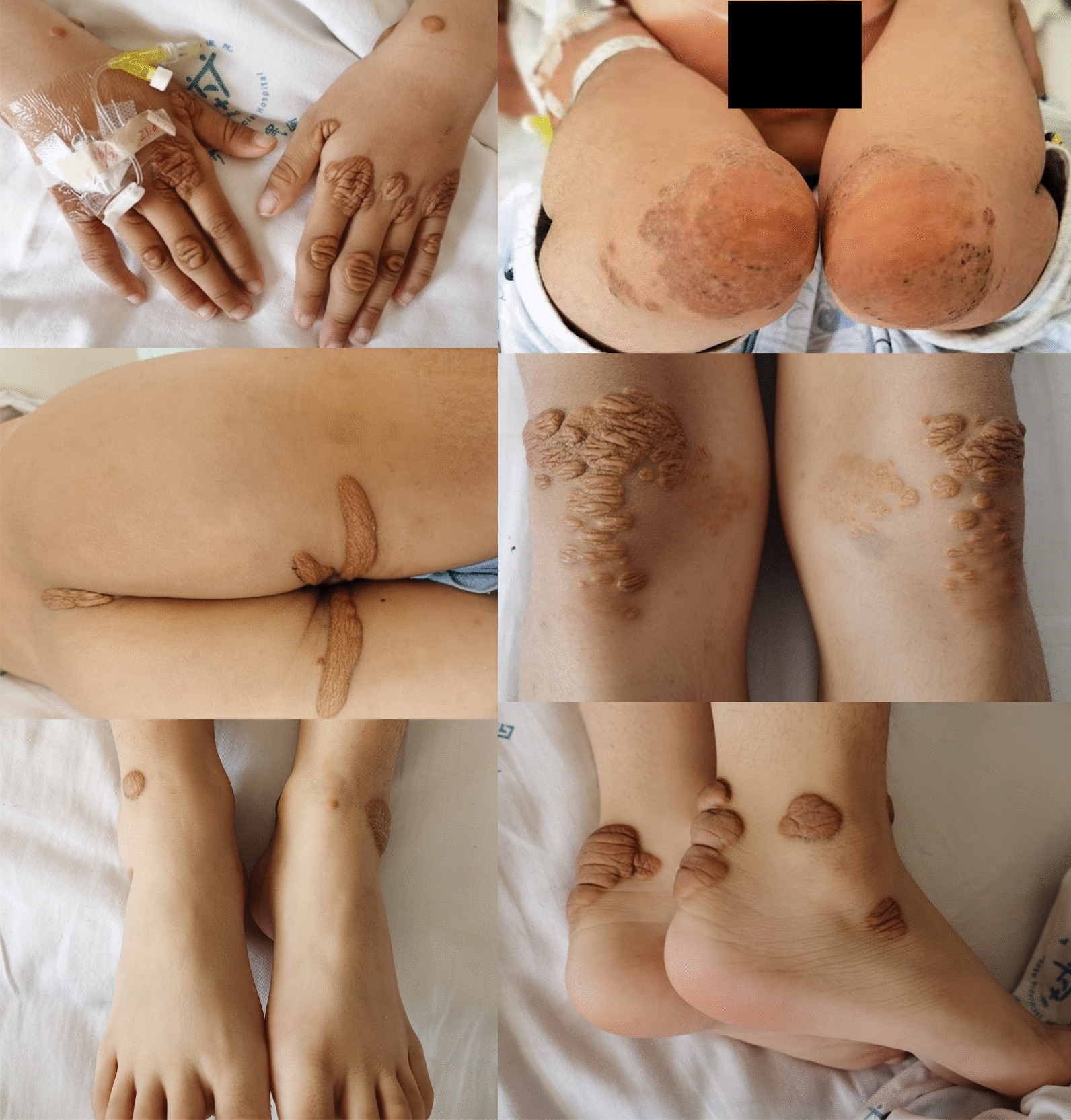
Photograph showing that skin xanthomas present on skin of both hands, both elbows, both knees, both ankles and the buttocks.

An 8-year-old Asian patient presented to our hospital with a diagnosis of ACS. At presentation, the patient had been having chest pain with slight exercise for 2 months. A 12-lead electrocardiogram (ECG) revealed sinus arrhythmia, electric axis deviation to the right and a roughly normal ECG (Fig. 1). Ischemic heart disease was suspected, and the patient was admitted to our hospital. On admission, his blood pressure was 110/60 mmHg, heart rate was 116 bpm and respiratory rate was 21 breaths per minute. His body height was 130 cm, body weight was 29 kg and body mass index was 17.16 kg/m^2^. The skin of both hands, both elbows, both knees, both ankles and the buttocks had a hemispherical yellow eminence subcutaneous nodules that ranged in size from 0.5 × 0.5 to 2.0 × 6.0 cm (Fig. 2). The boundary was clear, texture was tough and the surface was free from swelling and ulceration. Physical examination showed normal cardiac and pulmonary auscultation. The laboratory tests showed extremely high levels of blood lipids [triglycerides (TG) 1.89 mmol/l; total cholesterol (TC) 17.98 mmol/l; high-density lipoprotein (HDL) 0.83 mmol/l; LDL 14.02 mmol/l] (Table 1); N-terminal pro-brain natriuretic peptide (NT-proBNP) was 601 pg/ml, cardiac troponins T (cTnT) was 11.5 ng/l and C-reactive protein was 1.6 mg/l.

**Table 1:** Summary of the biochemical findings among the family members

Family member	Diagnosis	TG mmol/L	TC mmol/L	HDL-C mmol/L	LDL-C mmol/L
Proband	HoFH	1.89	17.98	0.83	14.02
Oldest sister	Normal	0.88	3.51	1.71	1.67
Second-oldest sister	HoFH	0.87	14.05	0.97	11.94
Third-oldest sister	HoFH	0.86	13.75	0.82	11.94
Fourth-oldest sister	HeFH	0.69	6.62	1.88	4.27
Fifth-oldest sister	HeFH	1.00	5.90	1.64	3.87
Father	HeFH	0.96	6.62	1.28	4.90
Mother	HeFH	0.95	6.64	1.16	4.74

*HeFH* Heterozygous familial hypercholesterolemia,* HoFH* homozygous FH

**Fig. 3:**
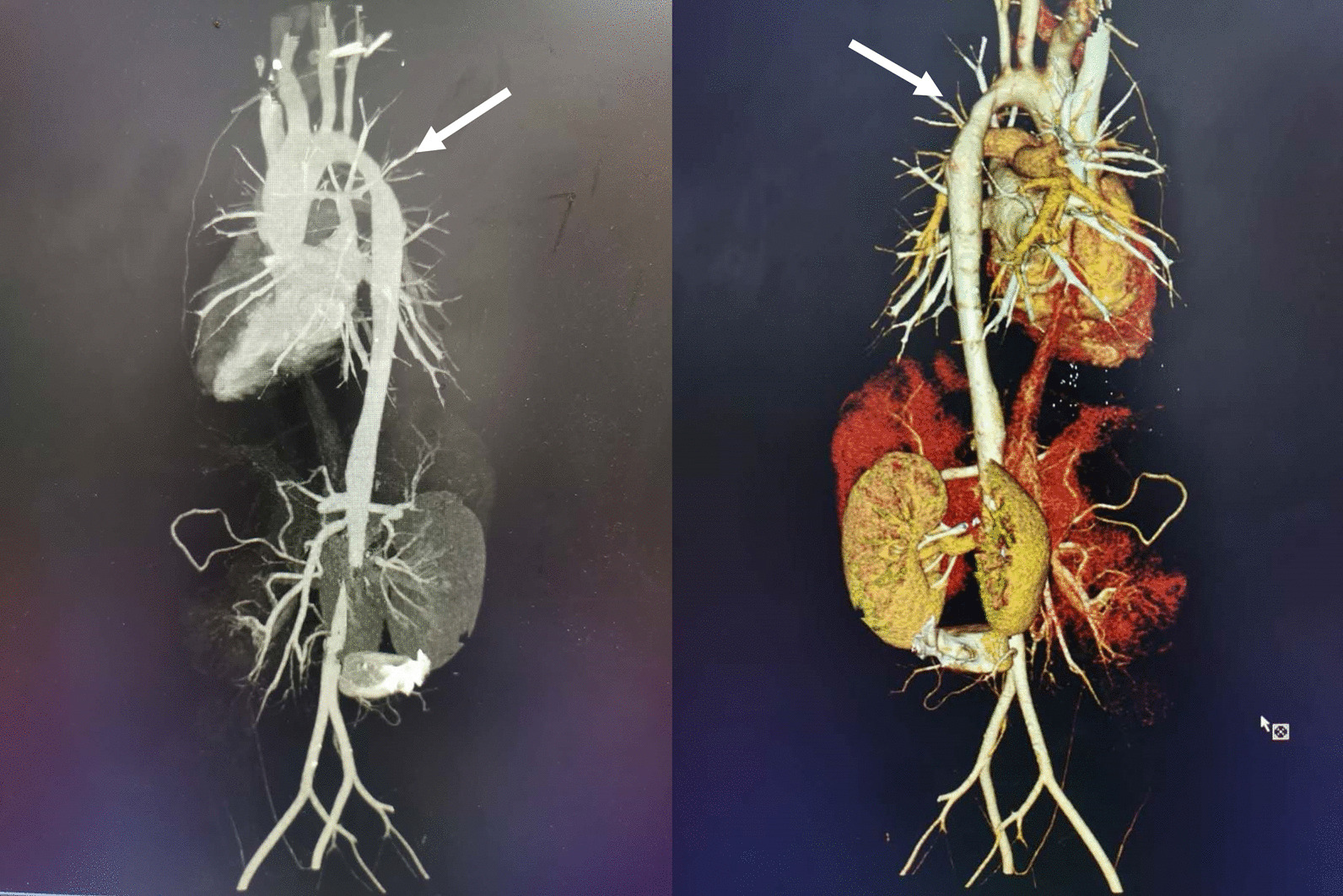
Computer tomography angiography showing stenosis of aortic arch of the patient (white arrow).

**Fig. 4:**
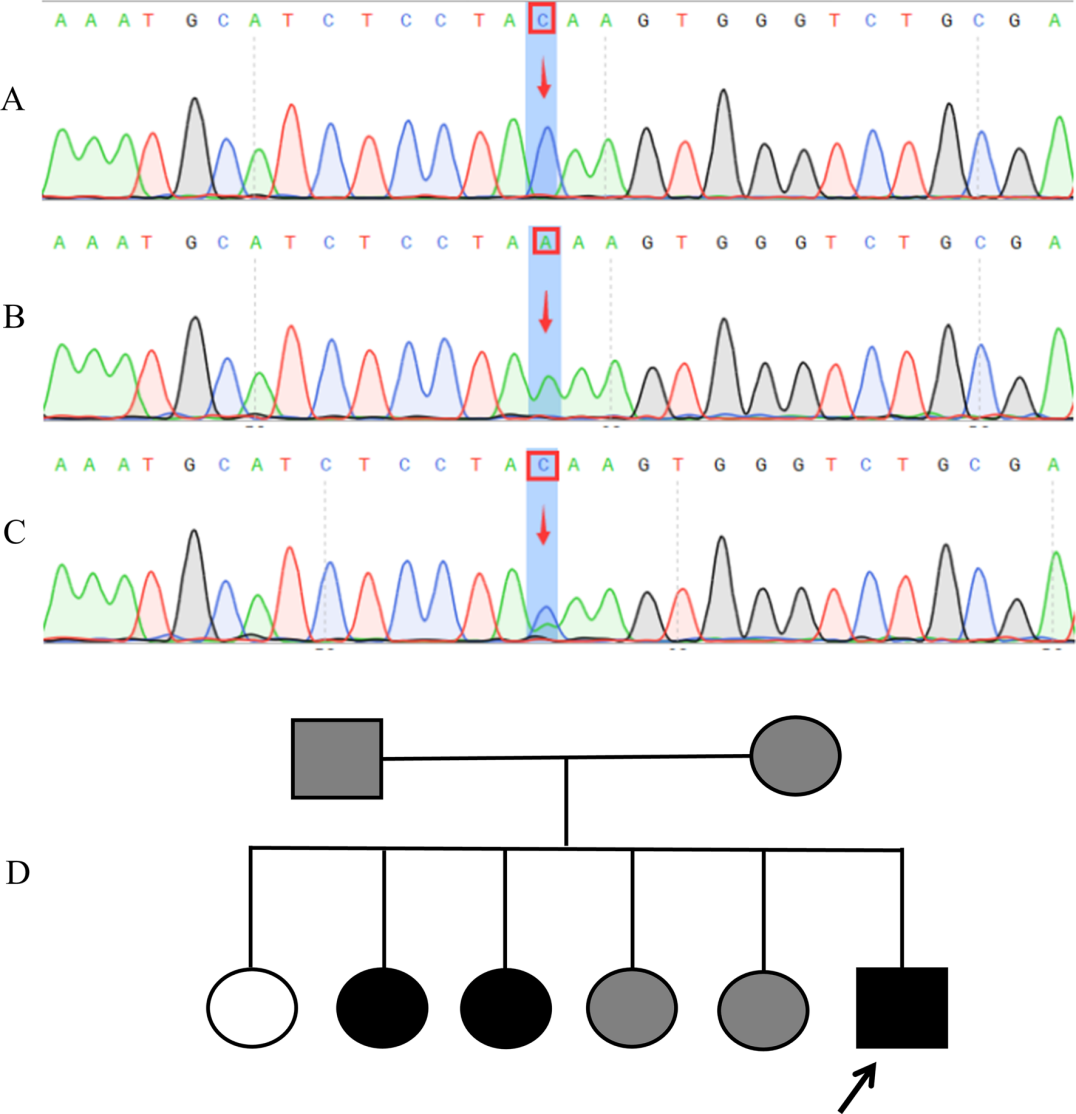
DNA sequencing results and the pedigree chart of the patient (proband) and family members. Shown here are sequencing histograms of the wild-type (**a**) and low-density lipoprotein receptor (*LDLR*) mutation DNA. Nucleotide CC at position 126 of exon 2 in the wild-type (arrow in **a**) had mutated to homozygous state nucleotide AA (arrow in **b**) and heterozygous state nucleotide CA (arrow in **c**). Based on the DNA sequencing results, a pedigree chart was established (**d**). Unfilled circle indicates a healthy female; gray square indicate a heterozygote male; gray circles indicate heterozygote females; black square indicates homozygous male; black circles indicate heterozygote females. The black arrow indicates the proband

Additional routine tests showed normal findings for blood, urine, fecal material, liver and kidney function, electrolytes, blood glucose and blood gas tandem mass spectrometry. ECG showed multiple pathological changes, including enlarged left ventricle, aortic sinus tube junction stenosis with mild regurgitation and mild mitral valve regurgitation. Chest computed tomography (CT) angiography showed stenosis in the aortic arch (Fig. 3), while CT of both lungs showed was normal. In view of the strong hypercholesterolemia and premature CAD, family cascade screening and genetic analyses were performed for the case and for both affected and unaffected family members, with the aim to detect the pathogenic FH mutations. Blood samples were acquired from the proband and the other seven family members, including the parents and the five older sisters. DNA sequencing of exons showed a C-to-A substitution at nucleotide 126 in exon 2, resulting in a mutation in the gene encoding the LDLR in the proband and in six other family members (Fig. 4). A representative sequencing histogram identifying the mutation and the pedigree chart created based on the DNA sequencing results is shown in Fig. 4.

**Fig. 5:**
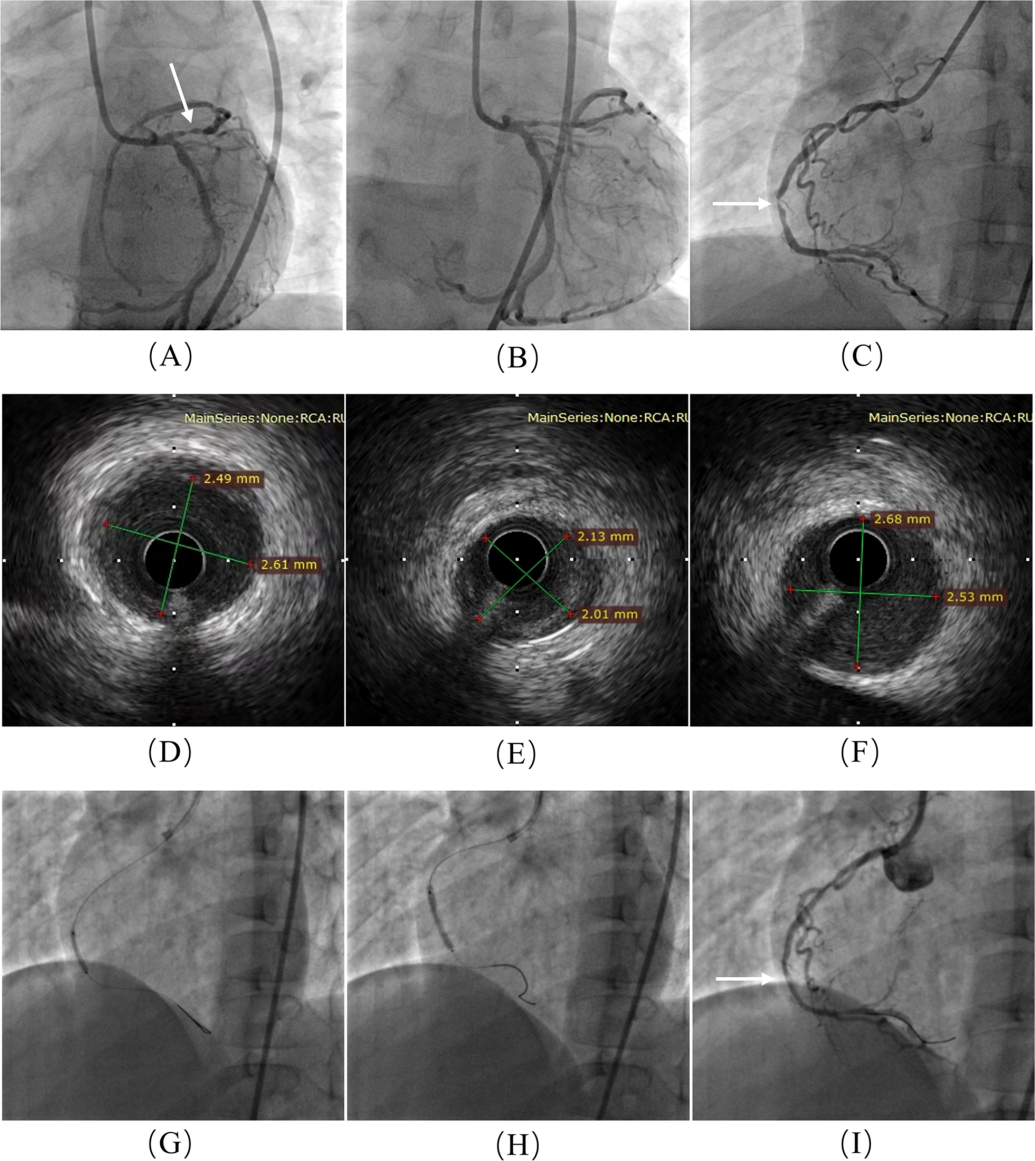
Coronary angiography (CAG). **a-b** 80–90% stenosis of the proximal left anterior descending coronary artery (white arrow). **c–f** 99% stenosis of the middle of the right coronary artery (white arrow) (**c**), with intravascular ultrasound showing that the diameter of the blood vessel in the normal segment near the stenosis is 2.49–2.61 mm (**d**), the diameter of the blood vessel at the location of highest stenosis is 2.01–2.13 mm (**e**) and the diameter of the blood vessel in the normal segment at the distal end of the stenosis is 2.53–2.68 mm (**f**). **g**, **h** Sprinter legend: 2.0 × 15 mm (**g**) and 2.5 × 20 mm (**h**). **i** Post-percutaneous transluminal coronary angioplasty, with excellent results (white arrow).

Based on the patient’s medical history, clinical manifestations, blood lipid levels and acute coronary symptoms, we diagnosed the patient with typical HoFH and ACS. Based on guidance from the 2014 AHA/ACC Guideline for the Management of Patients with Non-ST-Elevation Acute Coronary Syndromes (https://www.sciencedirect.com/science/article/pii/S0735109714062780?via%3Dihub) [7] and the Guidelines for Diagnosis and Treatment of Familial Hypercholesterolemia 2017 (https://www.ncbi.nlm.nih.gov/pmc/articles/PMC6099072/), we initiated the following treatments: (1) aspirin 50 mg taken orally (po), once daily (qd) and clopidogrel 50 mg, po, qd, to prevent thrombosis formation; (2) rosuvastatin 10 mg po, every night (qn) and diet control, to lower blood lipid levels; (3) ezetimibe tablets 10 mg po, qd, to stabilize plaque; (4) isosorbide dinitrate 2.5 mg po, twice daily (bid), to improve myocardial ischemia. Although the patient was taking rosuvastatin 10 mg and ezetimibe 10 mg daily, the level of LDL-C remained high (14.02 mmol/l). Therefore, we initiated double filtration plasma pheresis to reduce the level of LDL-C according the Guidelines for Diagnosis and Treatment of Familial Hypercholesterolemia 2017 [8]. Following this treatment, the child’s LDL-C level dropped to 3.18 mmol/l. CAG showed 80–90% stenosis of the proximal left anterior descending coronary artery (LAD) and 99% stenosis of the middle of right coronary artery (RCA), with the culprit identified as the RCA lesion (Fig. 5). Coronary bioresorbable stents were chosen as the best treatment considering the age of the patient. However, in our hospital, the normal diameter of the RCA of patients is 2.49–2.68 mm and the minimum diameter of the coronary bioresorbable stents is 2.75 mm. Therefore, we adopted the percutaneous transluminal coronary angioplasty (PTCA) strategy (Fig. 5). The patient’s angina symptoms improved significantly after PTCA, and he was discharged from our hospital.

## Discussion and Conclusions

Familial hypercholesterolemia is an autosomal hereditary disease. The most common cause of FH are mutations of the genes encoding lipid-handling proteins, such as LDLR, ApoB or PCSK9, which result in defects in LDL metabolism and, consequently, abnormally elevated plasma LDL-C levels [4, 8, 9]. FH can further be divided into HoFH and HeFH, with HoFH being the more serious and rare disease. Patients with HoFH usually die from coronary heart disease before the age of 30 years in the absence of any treatment [10]. The prevalence of HoFH is now thought to be between 1:250,000 and 1:500,000 persons based on the latest genetic studies [11]. The main clinical manifestations of HoFH are extremely high plasma LDL-C levels, corneal arch, tendon and skin xanthomas, premature coronary heart disease and aortic valve disease [12].

The 2017 Guidelines for Diagnosis and Treatment of Familial Hypercholesterolemia proposed that the diagnosis of HoFH should be based on: serum LDL-C level of ≥ 180 mg/dl; presence of xanthomas and atherosclerotic diseases from childhood; and parents who are HeFH [8]. The 2016 British HoFH management guide also recommends the use of genetic diagnostic or clinical diagnostic criteria for children and adults: (1) in children, LDL-C > 11 mmol/l and skin tendon xanthoma before 10 years of age; (2) in adults, LDL-C > 13 mmol/l and obvious xanthoma of skin tendon, or LDL-C level reaching clinical diagnosis standard, while parents were diagnosed as HeFH [13]. In accordance with these guidelines, our patient was diagnosed with HoFH.

Similar to the treatment of patients with HeFH, the current treatment options for HoFH include lifestyle interventions, such as dietary therapy, exercise therapy, smoking cessation and anti-obesity measures [14]. As atherosclerosis progresses more quickly in patients with HeFH than in their heterozygote counterparts, before providing guidance in exercise therapy and the patient initiating this exercise regimen, the patient should be carefully evaluated for CAD as well as valvular disease and aortic aneurysm [1]. In HoFH, ideal LDL-C management target levels are also < 100 mg/dl for primary prevention and < 70 mg/dl for secondary prevention. Even when treatment consists of multiple drugs combined with lifestyle intervention, it is still difficult to achieve LDL-C compliance in many patients with HoFH, which remains a serious hereditary disease [15]. The major mechanisms of action of statins, bile acid adsorbing resins and PCSK9 inhibitors all involve enhancing LDLR expression. In patients with the negative type, in which LDLR activity is completely absent, no LDL-C lowering effect is observed [16]. In this case, administration of PCSK9 inhibitors should be ceased if there is no decrease in LDL-C. However, PCSK9 inhibitors can only be used in patients aged > 12 years [17]. Nevertheless, for LDL-C control, LDL apheresis therapy once every 1–2 weeks is still required in many cases. When patients are resistant to all of the above treatments or show intolerance, liver transplantation may be considered [8].

The patient described here met multiple criteria of FH, including early-onset coronary heart disease, an extremely high plasma LDL-C level (14.02 mmol/l) and xanthoma formation on both hands, both elbows, both knees, both ankles and the buttocks. Genetic analysis showed that a C-to-A substitution at nucleotide 126 in exon 2 resulted in a mutation of the gene encoding LDLR in the proband and in six other family members, with the parents of the proband being heterozygous for the mutation and his two sisters being homozygous. This 8-year-old patient was definitely diagnosed with HoFH. Despite the high-intensity statin and ezetimibe treatments, the patient’s LDL-C were still high. Finally, filtration plasma was used to reduce the level of LDL-C, and PTCA improved the patient’s cardiac ischemia symptoms, enabling the patient to be discharged. Unfortunately, for patients from developing countries, continuous filtration plasma once every 2 weeks is not feasible. About 1 month later, the patient’s outpatient recheck LDL-C level reached 13.24 mmol/l.

In conclusion, we report here a child with ACS attributable to HoFH resulting from a mutation in the LDLR gene. To our knowledge, this is the youngest patient with a diagnosis of HoFH to be treated with PTCA after filtration plasma.

## Data Availability

All data will be available upon request made to the corresponding author.

## Abbreviations

ACS: Acute coronary syndrome
ApoB: Apolipoprotein B
CAG: Coronary angiography
cTnT: Cardiac troponins T
FH: Familial hypercholesterolemia
HDL-C: High-density lipoprotein-cholesterol
HeFH: Heterozygous FH
HoFH: Homozygous FH
LAD: Left anterior descending coronary artery
LDL-C: Low-density lipoprotein-cholesterol
LDLR: Low-density lipoprotein receptor
LDLRAP1: Low-density lipoprotein receptor adaptor protein 1
NT-proBNP: N-terminal pro-brain natriuretic peptide
PCSK9: Proprotein convertase subtilisin kexin-9
PTCA: Percutaneous transluminal coronary angioplasty
RCA: Right coronary artery
TC: Total cholesterol
TG: Triglyceride
